# The Low Prevalence of Inclusion Body Myositis in an Outpatient Rheumatology Myositis Cohort

**DOI:** 10.7759/cureus.9873

**Published:** 2020-08-19

**Authors:** Ehizogie Edigin, Ahmed S Hassan, Tanisha Mathur, Augustine Manadan

**Affiliations:** 1 Internal Medicine, John H. Stroger, Jr. Hospital of Cook County, Chicago, USA; 2 Internal Medicine, Healthlinc East Chicago, Indiana, USA; 3 Internal Medicine, The Valley Hospital, Ridgewood, USA; 4 Rheumatology, John H. Stroger, Jr. Hospital of Cook County, Chicago, USA

**Keywords:** inclusion body myositis, myositis, dermatomyositis, polymyositis

## Abstract

Introduction

Sporadic inclusion body myositis (IBM) is a rare type of myopathy of unknown etiology typified by the presence of distinctive muscle fiber inclusions. IBM belongs to a larger family of conditions called the idiopathic inflammatory myopathies (IIM). This study seeks to compare the prevalence, clinical manifestations, and disease course of IBM patients to a cohort of polymyositis (PM) and dermatomyositis (DM) patients in an outpatient rheumatology myositis cohort.

Methods

We conducted a retrospective chart review of all adult patients attending rheumatology clinics at the Cook County Hospital from 2006 to 2011 with the International Classification of Diseases, Ninth Revision, Clinical Modification (ICD-9) diagnoses of idiopathic inflammatory myopathies (IIM). Data collected included patient demographics, serial muscle strength testing, serial creatine kinase (CK), muscle biopsies, and immunosuppressive therapies received.

Results

We identified 112 patients with IIM with the following breakdown: 66 - dermatomyositis, 42 - polymyositis, and four - IBM. These four patients represent the primary cohort. They had a mean age of 67 years (range 62-74), the mean follow-up period of 15 months (range 3-47), mean initial muscle strength in the weakest group of 3.75 (range 2-5, standard deviation [SD]=1.3), mean initial CK of 1,968 U/L (range 313-4,795, SD=1,970), mean final muscle strength in the weakest group of 3.75 (range 3-4, SD=0.5), mean final CK of 1,326 U/L (range 213-2,943, SD=1,200).

Conclusions

Our IBM patients had many features similar to other described IBM cohorts, including older age, male predominance, slow progression (stable CK and muscle strength), and lack of response to immunosuppressive therapy. Interestingly, we found a low prevalence of IBM in our outpatient rheumatology IIM cohort.

## Introduction

Sporadic inclusion body myositis (IBM) is a rare type of myopathy of unknown etiology typified by the presence of distinctive muscle fiber inclusions [1]. IBM belongs to a larger family of conditions called the idiopathic inflammatory myopathies (IIM) which also includes dermatomyositis (DM) and polymyositis (PM). Patients with IBM classically have a phenotype significantly different from the other IIMs including older age at presentation, male predilection, insidious onset, combined distal and proximal muscle weakness, mixed neuropathic-myopathic electromyography (EMG), and lack of response to immunosuppressive therapy. IBM is recognized as the most common acquired IIM in patients over 50 years and has an estimated prevalence of 4.9-10.7 per million adults [1-3]. This study seeks to compare the prevalence, clinical manifestations, and disease course of IBM patients to a cohort of PM and DM patients in an adult outpatient rheumatology IIM cohort.

## Materials and methods

We conducted a retrospective chart review of all adult patients attending rheumatology clinics at the Cook County Hospital from 2006 to 2011 with a diagnosis of IIM. Institutional review board (IRB) approval was sought. However, since no patient identifiers were collected, the study was exempted from IRB review. Outpatient billing records were searched using the International Classification of Diseases, Ninth Revision, Clinical Modification (ICD-9) billing codes 359.71 for IBM, 710.3 for DM, 710.4 for PM, and 729.1 for myositis [4]. All relevant charts were accessed for review. Data collected included patient demographics, serial muscle strength testing, serial creatine kinase (CK), muscle biopsies, and immunosuppressive therapies received. DM and PM patients were categorized by the number of myositis criteria met, and IBM patients were categorized by meeting the clinical and histological criteria proposed by Lloyd, et al.: 1) finger flexor or quadriceps weakness; 2) endomysial inflammation, and 3) invasion of non-necrotic muscle fibers or rimmed vacuoles [5-9].

## Results

We identified 112 patients with IIM (66 - DM, 42 - PM, and four - IBM). Twenty-eight patients fulfilled criteria for definite PM and 14 for probable PM. Thirty-nine fulfilled criteria for definite DM and 18 for probable DM [2, 3]. Nine patients were classified as amyopathic DM. The details of the PM and DM patients have already been described [10]. Four patients were classified as IBM and represent the primary cohort. Three out of the four IBM patients were male, with a mean age of 67 years; range 62-74 (Table 1).

**Table 1:** Comparison of IBM with DM and PM patients IMB - inclusion body myositis; DM - dermatomyositis; PM - polymyositis; CK - creatine kinase, HMG-CoA - 3-hydroxy-3-methylglutaryl coenzyme A reductase

	IBM (n=4)	DM and PM (n=108)	p-value
Mean age (years)	67	45	0.001
Male sex (%)	75%	27%	0.034
Initial mean weakest muscle strength	3.75	3.6	0.795
Mean initial CK (U/L)	1,968	4,720	0.422
Final mean weakest muscle strength	3.75	4.6	0.013
Mean final CK (U/L)	1326	412	0.045
Mean follow up period (months)	15	62	
Received immunosuppression (%)	75	100	
HMG-CoA inhibitor exposure (%)	25	14	

The cohort consisted of two African-Americans, one Hispanic, and one caucasian. The mean follow-up period was 15 months (range 3-47). Mean initial muscle strength in the weakest group was 3.75 (range 2-5, standard deviation [SD]=1.3), mean initial CK was 1,968 U/L (range 313-4,795, SD=1,970), mean final muscle strength in the weakest group was 3.75 (range 3-4, SD=0.5), mean final CK was 1,326 U/L (range 213-2,943, SD=1,200). One out of four patients had dysphagia at presentation. Electromyography (EMG) showed a mixed neuropathic-myopathic pattern in three patients and purely myopathic in one patient. All four patients had muscle biopsies showing rimmed vacuoles consistent with a diagnosis of IBM. Electron microscopy was completed on three of muscle biopsy specimens and showed filamentous inclusions with whorled lamellar debris consistent with IBM. One patient was exposed to a 3-hydroxy-3-methylglutaryl coenzyme A reductase inhibitor (lovastatin), and one patient was exposed to ezetimibe prior to the diagnosis of IBM. 

During the follow-up period, three patients were treated with immunosuppression (prednisone, methotrexate, mycophenolate, and azathioprine) without improvement in muscle strength. All patients were alive at the end of the follow-up period.

The comparison between the two groups is outlined in table 1. At presentation, the IBM group was older (mean age 67.2 vs. 45.4 years; p=0.001) and had more males (75% vs. 26.6%, p=0.034) compared to the DM and PM group. However, both groups had similar initial CK levels (1,968 vs. 4,720; p=0.422) and mean initial muscle strength (3.75 vs. 3.63; p=0.795). At the end of the follow-up period, DM and PM group had significantly improved mean muscle strength (4.6 vs. 3.75; p=0.013) and mean CK (412 vs. 1,326 U/L; p=0.045) compared to the IBM cohort. The comparison of IBM to PM group > 50 years is outlined in Table 2.

**Table 2:** Comparison of IBM with PM patients above 50 years of age IMB - inclusion body myositis; PM - polymyositis; CK - creatine kinase

	IBM (n=4)	PM above 50 (n=16)	p-value
Mean age (years)	67	54	0.001
Male sex (%)	75%	31%	0.110
Initial mean weakest muscle strength	3.75	3.60	0.797
Mean initial CK (U/L)	1968	3414	0.470
Final mean weakest muscle strength	3.75	4.67	0.004
Mean final CK (U/L)	1326	360	0.005
Mean follow up period (months)	15	74	
Received immunosuppression (%)	75	100	

## Discussion

Data on the annual incidence of IBM is scarce and has varied considerably between studies, ranging from one to 70 cases per million adults [11-16]. This variation may be due to its insidious onset, which makes IBM a difficult diagnosis to make in a cohort of older patients where complaints of gradual non-specific weakness are common. IBM may, in fact, be underdiagnosed as it has been shown to be the most common IIM in adults over the age of 50 [13]. In one series, it was found that 86% of IBM patients who were mostly referred by neurologists were initially diagnosed incorrectly with other conditions [6]. There is also a long latency between IBM symptom onset and diagnosis of about six years [17-20]. This highlights the importance of more research to obtain a better estimate of the prevalence of IBM and a better awareness of its presenting symptoms. Men are more commonly affected, mean presenting age of 60 years (range of between 30-80 years), with very few patients with onset of symptoms before age 50 years [17-19].

The typical presentation of IBM is that of an insidious and progressive proximal leg weakness manifesting as difficulty climbing stairs, getting out of a chair, or repeated falls. IBM can also present with progressively worsening grip strength, decreased hand dexterity, or rarely with dysphagia as the only presenting symptom due to cricopharyngeal muscle involvement [11]. 95% of patients have distal finger flexor muscle weakness on physical examination. Most patients will also have weakness and atrophy of the quadriceps muscles [21, 22]. Contrary to DM and PM patients, both distal and proximal muscle groups are usually involved in IBM [21-23]. CK levels are either normal or mildly elevated, usually below 10 times the normal levels, and acute phase reactants, such as the erythrocyte sedimentation rate (ESR) and the C-reactive protein (CRP), are usually normal [19-24]. 

Like other described cohorts, our IBM patients had older age, male predominance, and slow progression (stable CK and muscle strength) and a mixed neuromyopathic pattern on EMG. Our PM and DM patients were younger, predominantly female, had an improvement in CK and muscle strength with immunosuppressive therapy, and had a pure myopathic pattern on EMG. In contrast to other cohorts, the mean initial CK in our IBM patients was greater than ten times the upper limit of normal [20]. Interestingly, we found a lower than expected prevalence of IBM in our IIM cohort. Only four out of 112 (3.6%) patients were ultimately determined to have IBM, while DM and PM were the overwhelming diagnoses in our IIM cohort. Even when we restricted our cohort to patients ≥50 years of age, PM and DM were still significantly more common than IBM (45 vs. 4 IBM patients).

To understand the low prevalence of IBM, we considered the possibility of misdiagnosis of IBM patients as PM. The absence of vacuoles on muscle biopsies does not fully exclude the diagnosis of IBM, especially in patients with a classic IBM phenotype. Around 15% of clinically defined IBM may not meet the histological criteria for diagnosis [25]. Although this consideration does not confound the diagnosis of DM due to the presence of the characteristic rashes, it may lead to the misdiagnosis of a small portion of IBM patients as PM. To overcome this issue, we separately analyzed the data for the subgroup of PM patients above the age of 50 years, which included 16 patients (Table 2). Of those patients, 13 had confirmed improvement or normalization of muscle strength with immunosuppression consistent with the diagnosis of PM. Of the remaining three patients, two had an unchanged muscle strength, and one patient did not have the necessary data available for comparison. The group of PM patients ≥50 years of age had a statistically significant difference in mean final weakest muscle strength (4.67 vs. 3.75, p=0.004) and a statistically significant lower final mean CK levels after immunosuppression (360 vs. 1,326 U/L, p=0.005) compared to the IBM group. Given these findings, it seems unlikely that biopsy-negative IBM accounts for the low prevalence of IBM in our IIM cohort. We postulate the low prevalence of IBM in our IIM cohort is related to the cohort being restricted to an outpatient rheumatology population.

Limitations of our study include the study design, which was a retrospective outpatient chart review of rheumatology clinic visits using ICD-9 codes. Hence, those patients who were seen in the neurology clinic but coded incorrectly, and inpatients were missed. Additional weaknesses include the small number of IBM patients and short follow up period. 

## Conclusions

Our IBM patients had many features similar to other described IBM cohorts including older age, male predominance, slow progression (stable CK and muscle strength), lack of response to immunosuppressive therapy, inclusions on muscle biopsy, and a mixed neuromyopathic pattern on EMG. In contrast to other cohorts, the mean initial CK in our IBM patients was greater than ten times the upper limit of normal, with a low prevalence of IBM among our outpatient rheumatology IIM cohort.
